# Influence of *Pseudomonas* sp. NEEL19 Expelled Volatile Compounds on Growth and Development of Crop Seedlings

**DOI:** 10.3390/microorganisms13122754

**Published:** 2025-12-04

**Authors:** Poovarasan Neelakandan, Fo-Ting Shen, Shih-Yao Lin, Shih-Han Lin, Chiu-Chung Young

**Affiliations:** 1 Department of Soil & Environmental Sciences, College of Agriculture and Natural Resources, National Chung Hsing University, Taichung 402, Taiwan; 2 Innovation and Development Center of Sustainable Agriculture (IDCSA), National Chung Hsing University, Taichung 402, Taiwan

**Keywords:** plant–microbe interaction, salt stress, VOCs, SPME-GCMS, DMDS, NH_3_, CO_2_, chlorophyll, stomatal cluster

## Abstract

This research intended to investigate the airborne chemical communication that occurs via volatile substances released by phyllosphere-associated bacteria, and it has been investigated whether it is beneficial to plants. The composition of halotolerant *Pseudomonas* sp. NEEL19 volatiles and impact on mung bean and fenugreek growth and metabolism were examined through co-culture in PPD. NEEL19 volatile mixtures (NEEL19 V^+^) enhanced the shoot and root length and chlorophyll content of mung bean under different saline conditions on short-term exposure. In particular, total chlorophyll a + b showed percentage increases of 58.15%, 67.00%, and 29.5% at 0, 50, and 100 mM NaCl, respectively. Furthermore, fenugreek seedlings’ biomass, shoot length, and chlorophyll content significantly increased while exposed to NEEL19 V^+^. In order to identify the range of volatile organic compounds (VOCs) that NEEL19 released, SPME-GCMS was utilized. The predominant VOC was dimethyl disulfide, while volatile inorganic compounds (VICs), including CO_2_ and NH_3_, were examined using the volatile trapping method. Saline stress of 100 mM NaCl influences the quantity and composition of both VOCs and VICs production in NEEL19. The consequences of aqueous NH_4_OH (1–5 μL) exposure seed PPD assay disclosed that NH_3_ is one of the responsible volatile substances that trigger substantial alterations in shoot length, root length, total chlorophyll, and stomatal structure in mung bean seedlings. Whereas, fenugreek seedlings exhibited a high chlorophyll content overall. This study indicates that the release of volatile mixtures from NEEL19 promotes the growth and development of mung bean and fenugreek seedlings.

## 1. Introduction

Bacteria are prokaryotic organisms that commonly reside in the rhizosphere, endosphere, and phyllosphere and interact with plants [1]. They can inherently modify the features and structure of their neighborhood, either positively or negatively, by producing secondary metabolites like endotoxins, biosurfactants, antibiotics, and airborne signaling molecules (volatiles). Plant growth-promoting rhizobacteria (PGPR) promote plant growth by fixing nitrogen, solubilizing minerals like phosphate, and synthesizing metabolites like auxin, cytokinin, 1-aminocyclopropane-1-carboxylate deaminase, and siderophores. The above-mentioned metabolites support plant growth by direct and indirect means and improve productivity under adverse environmental conditions like biotic and abiotic stress [2,3]. Among them, bacterial volatile compounds (BVCs) are the secondary metabolites released by PGPR, which are the least studied, yet they are new and a potential substitute for PGPR’s direct use. BVCs are microscopic effluvium composed of low-molecular-weight molecules that exhibit exceptional volatility and permeability in air and water (0.01 kPa at 20 °C) [4,5,6]. Because of their quick diffusion, BVCs behave like semiochemicals by undertaking chemical interactions with their neighborhood circumstances, such as fungus, insects, and plants, in both positive and negative manners [5].

BVCs are a diverse group of chemical compounds that comprises both organic and inorganic components [7]. Organic compounds are the primary products of many biosynthetic processes, including primary metabolism (sulfur) and secondary metabolism (fatty acids and terpenes), synthesized through the oxidation of carbohydrates. Diverse intermediate products include fermentation byproducts such as bacterial volatile organic compounds (VOCs), acids, terpenes, sulfur- and nitrogen-containing chemicals, and fatty acid derivatives (hydrocarbons, ketones, and alcohols) [8]. So far, more than 2000 VOCs have been reported from microbes [9]. Bacterial volatiles inorganic compounds (VICs) comprise carbon monoxide (CO), carbon dioxide (CO_2_), nitrogen (N_2_), ammonia (NH_3_), hydrogen cyanide (HCN), hydrogen sulfide (H_2_S), and nitric oxide (NO) [10,11].

Research has shown that diverse bacterial species, such as *Bacillus*, *Serratia*, *Arthrobacter*, *Stenotrophomona*, and *Pseudomonas* sp. strains, produce VOCs that are known to benefit various plant species at different growth stages, like biomass accumulation, shoot and root progression, chlorophyll content, and photosynthesis, as well as resilience to osmotic stress and drought, salt tolerance, and antifungal properties [12,13,14,15,16,17]. As an example, PGPR, which emits certain VOCs such as 2,3-butanediol, acetoin, dimethylhexadecylamine, and 2-pentylfuran, has been shown to promote plant growth by stimulating the enhancement of shoot, root, foliage, and photosynthetic pigments [18,19].

Other than VOCs, part of the VICs, such as CO_2_ and NO, also significantly enrich the development and vitality of plants [4]. Bacterial CO_2_ enhances plant development in closed co-culturing conditions [20,21]. Bacterial volatile HCN negatively affected plants, including *Arabidopsis thaliana*, lettuce, and Barnyard grass [22,23]. Furthermore, PGPRreleasing VICs enhances plant development either directly or indirectly. For instance, *Bacillus* sp. PG-8, *Bacillus licheniformis*, *Bacillus baekryungensis*, and *Chryseobacterium palustre* have exhibited the capacity to synthesize NH_3_, serving as a crucial nitrogen source for plants [24,25,26], and they also demonstrate antibacterial and antifungal properties against phytopathogens [27]. Therefore, both bacterial VOCs and VICs impact the growth and development of plants, although VICs have received less research attention than organic volatiles. Specifically, less research has been undertaken to establish the efficacy of VICs (like biogenic NH_3_) from bacteria on the growth of non-model plant species.

The phyllosphere contains fewer bacterial communities than the rhizosphere, but some of these strains have characteristics that can enhance plant growth (synthesis of phytohormone and siderophore), comparable to PGPR. In that manner, phyllosphere-associated bacteria release a variety of VOCs, which can substantially modulate plant physiology and evolution to environmental stress adaptation [28]. Whereas, compared to phyllosphere isolates, major former research on VOCs’ ability to interact with plants has employed bacterial strains associated with roots. Hence, conceptual underpinning is still required for comprehending how phyllosphere bacterial populations modulate plants through volatiles under conditions resembling environmental stress to gain a better understanding.

In this research, we hypothesize that VOCs and VICs produced by phyllosphere-associated bacteria could induce the growth of crop plant seedlings under saline stress. This hypothesis was investigated using the halotolerant-phylloplane isolate, *Pseudomonas* sp. NEEL19 volatiles biostimulating characteristics and growth enhancement of mung bean and fenugreek seedlings. Additionally, the interaction of biogenic NH_3_, which is a crucial VIC emitted by the NEEL19, inducing growth enhancement in mung bean and fenugreek seedlings, was examined.

## 2. Materials and Methods

### 2.1. Halotolerant Bacterial Strain and Seeds

NEEL19 was cultivated in nutrient broth with a prescribed dose of NaCl (0, 2, 5, 6, 8, and 10%; *w*/*v*). About 4 mL of media in 20 mL test tubes were inoculated and incubated with cells for 72 h at 30 °C and agitation of 150 rpm in triplicate. Every 6 h, OD_600_ was measured using the Ultrospec 10-cell density meter (Amersham Biosciences, Woburn, MA, USA). In addition, a phosphate solubilization assay was performed for the NEEL19 strain [29]. Seeds of mung bean (*Vigna radiata* L.) and fenugreek (*Trigonella foenum-graecum* L.) (purchased local market) were surface sterilized by soaking them in 70% ethanol for 30 s, followed by 2% sodium hypochlorite for 5 min. The seeds were rinsed four to five times with ddH_2_O and soaked with sterile ddH_2_O for 7 h, and were utilized for further experiments.

### 2.2. Growth Analysis of Mung Bean and Fenugreek Seedlings Under NEEL19 (NEEL19 V^+^) Volatile Exposure

The cotton bed (approximately 0.25 g) with water (3.5 mL of sterile ddH_2_O) possessing different saline concentrations (0, 50, and 100 mM of NaCl) was prepared for the surface-sterilized mung bean seeds on one part of the partition petri dish (PPD). Whereas, nutrient agar (NA) possessing different saline concentrations (0, 50, and 100 mM of NaCl) was spot inoculated with a cell suspension of NEEL19 (20 μL of 6.1 × 10^6^ CFU mL^−1^) on the other part. Uninoculated NEEL19 NA plates (NEEL19 V^−^) were used as controls at each NaCl concentration. The analysis was performed in triplicate (a total of 15 seedlings for each treatment). Then, the PPD was securely sealed with insulating polyvinyl chloride (PVC) tape and incubated for 7 d at 25 ± 2 °C with a photoperiod of 5 d in the dark and the final 2 d in 8 h of light and 16 h of darkness. Furthermore, an examination of fenugreek seed growth was conducted using a method identical to mung bean without salt stress.

### 2.3. NEEL19 Volatilome Investigation

#### 2.3.1. Identification of NEEL19 VOCs by SPME-GCMS

The extraction of VOCs from the bacteria was performed based on the procedure, with a few modifications [30]. About 100 µL of bacterial culture was inoculated with 20 mL of NA medium (with or without 100 mM NaCl) in a 125 mL Erlenmeyer flask. The control used here is the blank NA medium (with or without 100 mM NaCl). Then the inoculated flask was secured well with parafilm and allowed to grow for 5 d at 30 °C. SPME needle (filled with 75 µm CAR/PDMS SPME fiber (Supelco, Bellefonte, PA, USA) was prepared and conditioned for 30 min at 280 °C in advance with helium gas. The prepared needle was inserted through the parafilm layer, which was held undisturbed to absorb the VOCs released by the bacteria for 12 h, which are accumulated in the flask’s headspace. GC-MS was employed to ascertain the presence and composition of VOCs. Following the extraction of VOCs, the SPME fiber was introduced into the injection port of the GC-MS instrument. The specific equipment used was the QP2010 SE model manufactured by Shimadzu Corporation in Kyoto, Japan. The GC-MS instrument was equipped with an RTx-5MS column, which had dimensions of 30 m in length, 0.25 mm in diameter, and a thickness of 0.50 µm. The carrier gas utilized in the experiment was helium, with a flow rate of 1 mL min^−1^. The temperature settings for the GC oven were as follows: an initial temperature of 40 °C for 5 min, followed by a gradual increase from 40 to 120 °C at a rate of 3 °C per min. Subsequently, the temperature increased from 120 to 180 °C at a rate of 4 °C per min. Further, the temperature was raised from 180 to 280 °C at a rate of 20 °C per min and maintained at 280 °C for 5 min. The identification of VOCs was proposed through a comparative analysis of the substance’s mass spectrum with the database of the National Institute of Standards and Technology (NIST20) [31] using GC/MS technology. The bacterial cultures were subjected to a minimum of two experimental trials.

#### 2.3.2. Assessment of VICs Released by NEEL19

PPD was set up with a compartment holding a spot inoculation of NEEL19 possessing different saline concentrations (0, 50, and 100 mM of NaCl) on NA and placed a 1.5 mL microfuge tube with 1 mL of sterilized ddH_2_O in the other compartment to trap the released volatile trapped water-VTW) and NEEL19 free NA plates were kept as controls. For every 24 h until 7 d, at 30 °C of incubation, NEEL19-generated VTW was retrieved from PPD, followed by the ammonia quantification (colorimetric) assay, as illustrated by Abdelwahed, et al. [32]. A total of 50 µL of VTW was combined with 100 µL of Nessler’s reagent, the trapped NH_3_, incubated for 10 min at room temperature, and read at 450 nm (CLUBIO (AMR-100) Hangzhou Allsheng Instruments Co., Ltd., Hangzhou, China). Ammonium sulfate was used as a standard range between 0.5 µM–4 mM mL^−1^.

With a few adaptations, the CO_2_ trapping experiment was carried out as described [21,33]. NEEL19 was spot-inoculated on NA amended with NaCl (0, 50, and 100 mM) on one side, and 0.5 g of Ba (OH)_2_.8H_2_O powder on the opposite side; then, the PPD was sealed snugly with PVC tape, including NEEL19-free NA plates as a control. The experiment was then incubated for 7 d at 30 °C. BaCO_3_ was formed upon interaction between the gaseous CO_2_ released by NEEL19 and Ba (OH)_2_.8H_2_O in PPD. The insoluble BaCO_3_ was filtered and measured gravimetrically in triplicate.

NEEL19 produced VTW was used in quantifying ammonium carbonate (NH_4_)_2_CO_3_, following the acid–base titration method [34]. In short, the ammonium carbonate and bicarbonate mixture released from NEEL19 gets dissolved in 1 mL of VTW water. The VTW was mixed with 20 µL of 1% phenolphthalein, and a pink color developed. The pink color vanished when titrated against 0.1 M HCl, which is the ammonium carbonate solution’s endpoint of acidity/alkalinity of VTW was investigated by pH-Fix indicator strip (Macherey-Nagel-ref-92118).

### 2.4. Analysis of Mung Bean and Fenugreek Growth in PPD Exposed to Aqueous Ammonium Hydroxide Vapor (NH_4_OH)

To assess the impact of specific volatile compounds on mung bean a growth under the PPD system previously described, five seeds were put on the cotton bed compartment, and different dosages of aqueous ammonium hydroxide (28% *v*/*v*), ranging from 1, 2, 3, 4, and 5 μL in the 500 μL microfuge tubes, were placed on the opposing side of the PPD. About 5 μL of sterile ddH_2_O was kept as a control. The PPD was wrapped in PVC tape and incubated for 7 d at 25 ± 2 °C with a photo period of 5 d in the dark and the final 2 d in 8 h of light and 16 h of darkness. From triplicates (a total of 15 seedlings for each dosage), measurements were made on the plant growth characteristics. Additionally, fenugreek seed growth was examined using the same methodology as mung beans.

### 2.5. Chlorophyll Content Measurement

The chlorophyll content for NEEL19 V^+^ and NH_4_OH-exposed mung bean and fenugreek seedlings were observed. The grinding–settling technique, with the following changes, was used to extract the chlorophyll [35]. About 0.2 g of fresh cotyledonary leaves was mashed for 2 min in a mortar and pestle containing 5 mL of 80% acetone, and the ground mixture was subsequently left overnight at 5 °C. On the following day, the extract was measured at 645 and 664 nm (Biochrom Asys UVM 340, Biochrom Ltd., Cambridge, UK). As for the fenugreek seedling, 0.1 g of fresh cotyledon was added to 2.5 mL of 80% acetone. The concentrations of total chlorophyll, chlorophyll a, and chlorophyll b were determined for triplicate samples (in total of 15 seedlings in each treatment).

### 2.6. Morphometric Analysis of Mung Bean Cotyledonary Leaf Stomata

Several treatments were investigated, including NEEL19 V^+^ and NH_4_OH, to understand the changes that occur in the morphology of stomata. With little modification in the methodology of Hosseini, et al. [36], the analysis was performed. A thin layer of adaxial face epidermis was peeled from cotyledonary leaves, and the epidermis samples were mounted on tiny glass slides. Further examination was done with a light microscope (AXEN/SP301C, East Tree, Ltd., Hsinchu, Taiwan). Images (100 and 400× magnification) were recorded by an AXIOCAM-ZEISS 105 color camera (ZEISS, Oberkochen, Germany). Measurements of stomatal density, length, and width were analyzed by Axiovision (AxioVs X 64 V4.9.1.0) software. All treatments were performed in triplicate (for each image treatment with three cotyledonary leaves, a total of 40 × 3 = 120 stomata. Closed stomatal proportion was also measured.

### 2.7. Statistical Analysis and Software

Significant differences on quantified NEEL19 volatiles (NEEL19 V^+^) under saline stress were assessed using a t-test, while the NH_4_OH vapor exposure analysis was followed with Duncan’s method. Statistical analysis was performed using SPSS 21.0 software and figures were generated using GraphPad Prism 6.

## 3. Results

### 3.1. The Influence of NEEL19 V^+^ on the Development of Mung Bean and Fenugreek Seedlings

**Figure 1:**
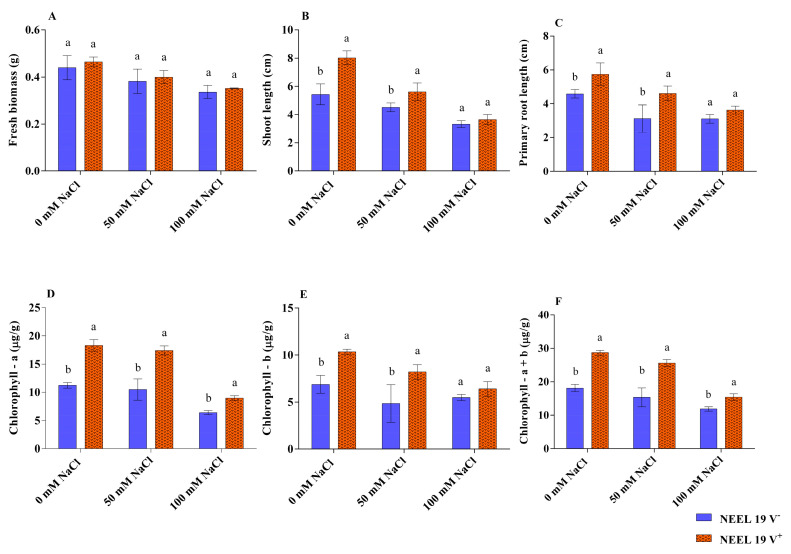
Effect of NEEL19 V^−^ and V^+^-exposed mung bean seedlings under different saline conditions (0, 50, 100 mM NaCl). (**A**) Biomass, (**B**) shoot length, (**C**) root length, (**D**) chlorophyll a, (**E**) chlorophyll b, and (**F**) total chlorophyll a + b. Significant differences (ANOVA followed by Tukey’s test, *p* < 0.05) among treatments are indicated by letters (n = 3).

NEEL19 exhibited saline tolerance ability up to 6% NaCl in the nutrient broth, which was further utilized in this research (Figure S1A). NEEL19 showed a positive sign of phosphate solubilization in the tricalcium phosphate agar plate with solubilization index (PSI) is 0.37 ± 0.06 (Figure S1B). Seven days after exposure to varying saline concentrations (0, 50, and 100 mM NaCl), volatile compounds from NEEL19-exposed seedlings (NEEL19 V^+^) were collected, along with a control (NEEL19 V^−^) (Figure S2A–F). In the PPD, no discernible difference in the biomass was observed in all NaCl concentrations compared with their respective control (Figure 1A), whereas enhanced shoot length and root length were found to be significant compared to their respective NEEL19 V^−^ at a concentration of 0 (47.7% and 25.3%) and 50 mM (24.4% and 47.8%), but not in 100 mM (Figure 1B,C).

Chlorophyll content was also analyzed resulting NEEL19 V^+^ under 0 mM NaCl treatment, showing an overall increase in chlorophyll a, chlorophyll b, and total chlorophyll a + b production with the concentration of 18.31 μg/g, 10.40 μg/g, and 28.67 μg/g, respectively, with a percentage increase of 62.79%, 51.10%, and 58.14% (Figure 1D–F). Saline-exposed seedlings with NEEL19 V^+^ showed a significant increment in chlorophyll a and total chlorophyll (a + b) compared to their respective NEEL19 V^−^. Particularly, NEEL19 V^+^-exposed total chlorophyll under saline stress (50 and 100 mM), exhibiting percentage enhancements of 67.00% and 29.57%, respectively. Like biomass, shoot length, and root length, saline-treated seedlings NEEL19 V^−^ showed lower chlorophyll production than 0 mM NaCl NEEL19 V^−^, resulting in a gradual diminishing growth with the increasing NaCl concentration. Furthermore, fenugreek seedlings exposed to NEEL19 V^+^ showed notable differences in biomass and shoot length as compared to the NEEL19 V^−^ (control) (Figure S5A,B). Interestingly, NEEL19 V^+^ exposed fenugreek seedlings’ chlorophyll (chlorophyll a, b, and total chlorophyll a + b) was studied and found a significant increase in percentages of 33.27%, 25.72%, and 29.80%, respectively, compared to non-exposed NEEL19 V^−^ controls (Figure S5D–F). We observed that the NEEL19 V^+^-exposed fenugreek seed coat color shifted from yellow to greenish (Figure S4A,B). The findings of this study demonstrate that the volatile compounds emitted by the NEEL19 play a crucial role in mitigating the adverse effects of saline stress on plant growth, hence facilitating its overall development. The growth promotion data demonstrated that volatiles formed by NEEL19 may have significant implications in stimulating plants’ growth and development.

### 3.2. Volatilome Analysis of NEEL19

#### 3.2.1. VOCs Profiling

**Figure 2:**
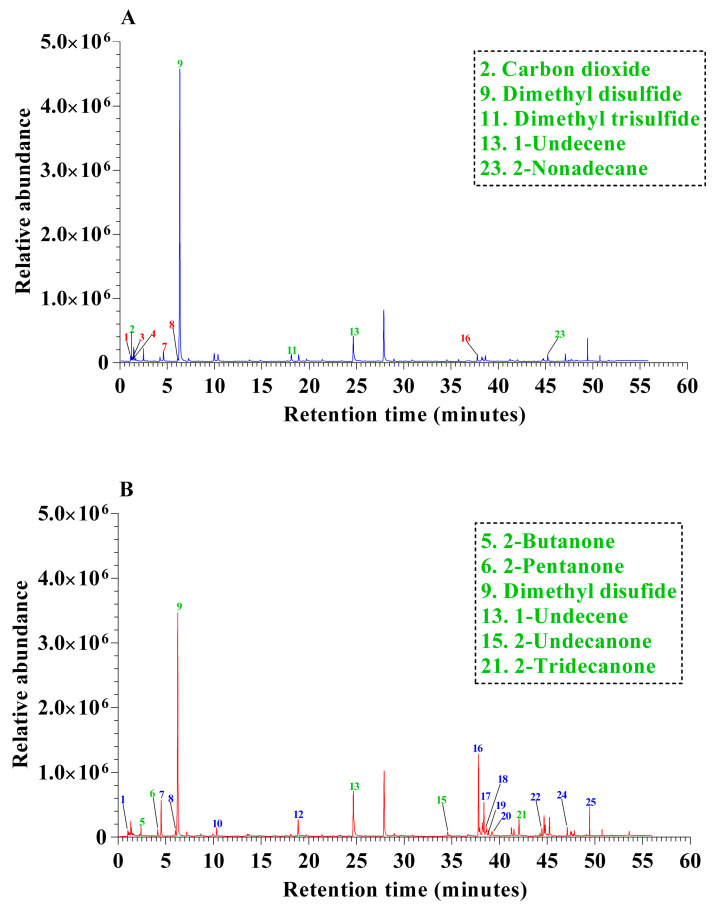
Chromatographic profile of VOCs released by NEEL19 in NA medium under saline conditions, (**A**) 0 mM and (**B**) 100 mM NaCl. The numbers indicated in the graph represent the peak number. Green colour numbers indicate the only VOCs involved in the plant growth promotion in (**A**,**B**).

Organic volatiles released by the NEEL19 are identified by running SPME-GCMS. The chromatographic profile showed 12 peaks in 0 mM NaCl (Figure 2A) and 19 peaks in 100 mM NaCl (Figure 2B) saline stress conditions, which include acid, amide, alkene, alcohol, ketone, hydrocarbons, and sulfur-containing compounds (Table S1).

The NEEL19 VOCs profile exhibited 7 distinct organic substances in 0 mM NaCl and 13 distinct organic substances under 100 mM NaCl saline stress conditions. Additionally, five comparable VOCs were found in both treatments. The highest peak was dimethyl disulfide (DMDS) found (61.75%) in 0 mM NaCl, compared to 32.78% in 100 mM NaCl. Notably, for methyl thiolacetate, 1-undecene peak areas were increased (1.49% and 5.71% in 0 mM NaCl, whereas 3.61% and 8.74% in 100 mM NaCl) under saline conditions compared to 0 mM NaCl-inoculated NEEL19. Methanethiol (0.11%) in 0 mM NaCl and 2-Undecanone (0.99%) in 100 mM NaCl provided that NEEL19 possessed the lowest area peak. Based on previous references, NEEL19-released plant-beneficial VOCs are notified in the green color in Table S1 [37,38,39] and their corresponding mass spectrometry data are shown in Figure S3.

#### 3.2.2. Analysis of VICs

**Figure 3:**
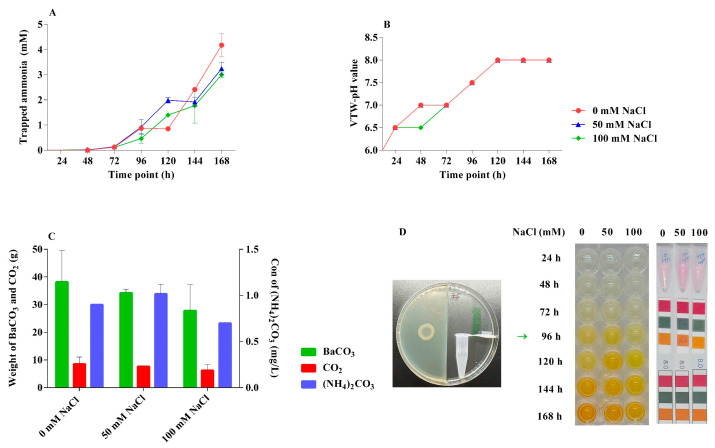
Quantification of VICs emitted from NEEL19. (**A**) Quantification of trapped ammonia in 1 mL of ddH_2_O by VTW method. (**B**) pH of the trapped VTW. (**C**) Amount of CO_2_ and (NH_4_)_2_CO_3_ released by NEEL19 in PPD under different NaCl concentrations (0, 50, 100 mM). (**D**) Pictorial representations of VTW method, estimation of trapped ammonia, and pH of the VTW.

NEEL19 was detected to emit diffused inorganic volatiles such as NH_3_, CO_2_, and (NH_4_)_2_CO_3_ by the trapping method under different saline (0, 50, 100 mM NaCl) stress conditions (Figure 3). The biogenic volatile NH_3_ was released from NEEL19 and allowed to interact with the ddH_2_O on the other side of PPD to generate VTW NH_3_ and create an equilibrium of NH_3_–NH_4_^+^. With the addition of Nessler’s reagent, VTW NH_3_ was both verified and estimated. Based on the concentration of NH_3_, a color transition from light yellow to deep yellow to brownish took place (Figure 3D), but the control (non-inoculated NEEL19 plates) showed no color change. After 24 h of incubation, it was seen that NEEL19 was emitting NH_3_ in all treatments (0, 50, 100 mM NaCl). Following measurements of the trapped NH_3_ every 24 h up to 168 h, different polynomial trendlines were produced (Figure 3A). At 168 h, the highest concentration of released NH_3_ was 4.17 mM (0 mM NaCl), followed by 3.24 mM (50 mM NaCl) and 3.00 mM (100 mM NaCl). Acidity/alkalinity of VTW was also determined using a rapid test strip. In comparison to the control, the alkalinity of VTW dramatically increased to pH = 8 after 120 h (Figure 3B) in all treatments (0, 50, 100 mM NaCl).

CO_2_ released by NEEL19 reacts with the Ba(OH)_2_ installed on the other side of the PPD, yielding insoluble BaCO_3_. On NEEL19 exposure at 168 h, the insoluble BaCO_3_ was observed to be considerably higher, with 38 mg generation in 0 mM NaCl in comparison to the 50 and 100 mM NaCl saline stress conditions (Figure 3C). High CO_2_ (8.5 mg) was found in NEEL19 (0 mM NaCl), while the lowest CO_2_ (6.2 mg) was recorded in 100 mM NaCl. NEEL19 emitted CO_2_ was reduced while increasing the concentration of saline stress. In addition, VTW was utilized to analyze the concentration of (NH_4_)_2_CO_3_ by acid-base titration. In all treatments (0, 50, 100 mM NaCl) exhibited presence of (NH_4_)_2_CO_3_ compared to the control (non-inoculated NEEL19 plates). Particularly, the highest concentration of (NH_4_)_2_ CO_3_ (1.01 mg/L) was detected in 50 mM NaCl, whereas the lowest value (0.7 mg/L) was in 100 mM NaCl (Figure 3C).

### 3.3. Influence of Mung Bean and Fenugreek Growth upon Aqueous NH_4_OH Exposure in PPD

**Figure 4:**
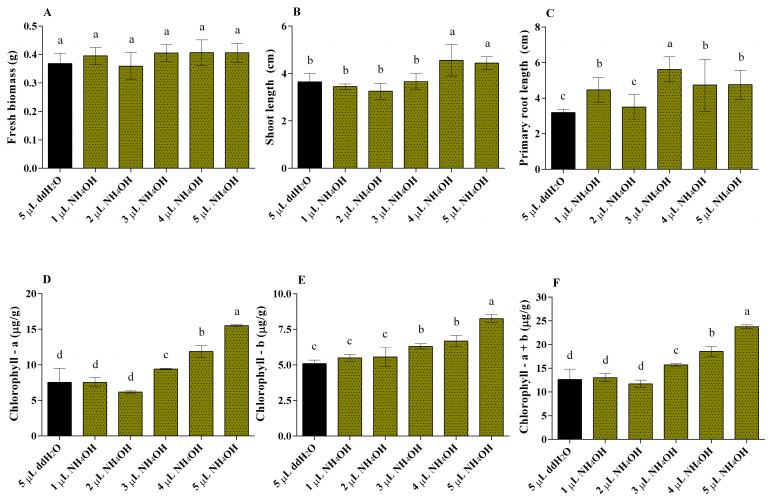
Assessment of the aqueous ammonia vapors (1 to 5 µL NH_4_OH) dose-dependent growth analysis on mung bean seedlings. (**A**) Fresh biomass, (**B**) shoot length, (**C**) root length, (**D**) chlorophyll a, (**E**) chlorophyll b, (**F**) total chlorophyll a + b. About 5 µL ddH_2_O was used as a control. Significant differences (ANOVA followed by Duncans test, *p* < 0.05) among treatments are indicated by letters (n = 3).

NH_4_OH was employed to find a comparable growth boost when NH_3_ was shown to be one of the probable NEEL19-volatile growth inducers. In a PPD, seedlings of mung bean on the one hand, and various concentrations (0, 1, 2, 3, 4, and 5 µL) of aqueous NH_4_OH in a microfuge tube on the other part was tested (Figure S2G–L). An increase in the biomass concentration of 3, 4, and 5 µL of NH_4_OH was observed, it was not statistically. Meanwhile, significant results for shoot length enhancement were obtained for 4 and 5 µL with 4.60 cm (25.87%) and 4.44 cm (21.75%), respectively. When compared to unexposed mung bean seedlings to NH_4_OH vapors, a substantial increase in root length was seen on 1 µL (39.66%), 3 µL (76.82%), 4 µL (48.22%), and 5 µL (48.85%) (Figure 4A–C).

The maximum root length was exhibited in 3 µL NH_4_OH-exposed seedlings about 5.64 ± 0.47 cm. All the NH_4_OH-exposed seedlings displayed the formation of a lateral root that resembled that of NEEL19-exposed seedlings (Figure S2). Furthermore, photosynthetic pigments (chlorophyll a, chlorophyll b, and total chlorophyll a + b) were also estimated. The amount of chlorophyll a, b, and total chlorophyll a + b was significantly increased in 3, 4, and 5 µL of NH_4_OH-exposed mung bean seedlings by chlorophyll a 24.40%, 56.78%, and 105.21%; chlorophyll b 23.84%, 31.16%, and 61.81%, and total chlorophyll a + b 24.18%, 46.46%, 87.73%, respectively, compared to the control. The maximum amount of total chlorophyll a + b content was found to be 23.75 ± 0.39 μg/g in 5 µL NH_4_OH-exposed mung bean seedlings (Figure 4D–F). In addition, fenugreek seedlings showed that the amount of chlorophyll a significantly increased in 3 and 4 µL of NH_4_OH treatment by 39.82% and 19.29%. The percentage increase of chlorophyll b 7.38%, 21.50%, and 23.37%, and total chlorophyll a + b 24.87%, 20.30%, and 16.33%, respectively, increased in NH_4_OH (3, 4, and 5 µL)-exposed fenugreek seedlings compared to the control. The maximum amount of total chlorophyll a + b content was found to be 29.95 ± 0.48 μg/g in 3 µL NH_4_OH exposed fenugreek seedlings (Figure S6D–F). The biomass, shoot length, and root length do not significantly differ from one another, and we found that the seed coat color changed from yellow to green after being exposed to 2–5 µL of NH_4_OH (Figure S4E–H).

### 3.4. Morphological Alterations in the Seedling’s Stomata of Mung Bean Subjected to NEEL19 V^+^ and NH_4_OH

**Figure 5:**
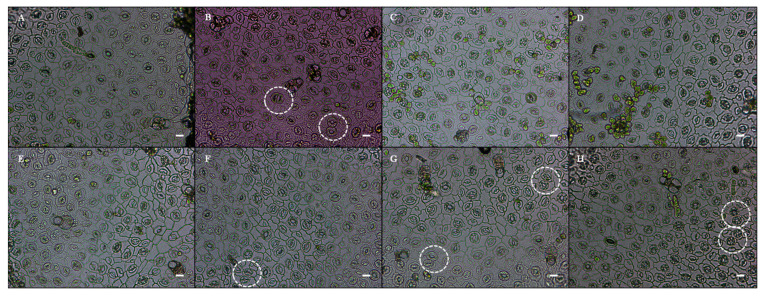
Light microscopic stomatal morphologic observations of mung bean seedlings exposed to (**A**) NEEL19 V^−^ and (**B**) NEEL19 V^+^ and the exposure of aqueous ammonia vapors. (**C**) About 5 µL of ddH_2_O was used as a control. (**D**–**H**) 1 to 5 µL of NH_4_OH on PPD, respectively. The white circle indicates a putative contiguous stomatal cluster and bars = 20 μm.

Morphological features such as stomatal density, size, and closure were analyzed for NEEL19 V^+^ and NH_4_OH exposure. The mung bean seedlings in the closed PPD plates produced a distinct leaf stomatal structure (with one or two putative stomatal clusters) after being exposed to NEEL19 V^+^ (Figure 5B) and 3–5 µL of NH_4_OH (Figure 5F–H), but not in the control. Additionally, the measurement of stomatal density involved counting the stomata. Seedlings that were exposed to NEEL19 V^+^ showed 16.15% increased density than the control (NEEL19 V^−^) group. Additionally, a considerable percentage increase of around 6.39% and 4.60% in terms of stomatal length and width was also observed. The proportion of closed stomata is another factor. When compared to the control, seedlings exposed to NEEL19 V^+^ demonstrated an increase in stomata closure of 97.01% (Table 1). This demonstrated that the NEEL19 V^+^ exposure enhanced the length and breadth of stomata in addition to their quantity and closure.

As for NH_4_OH (1–5 µL), in comparison to seedlings with 0 µL added ddH_2_O, the results revealed a stomatal density and closure percentage significant rise of 19.01%, and 57.75% in 3 µL; 13.39%, and 57.75% in 4 µL; and 11.26%, and 54.93% in 5 µL, respectively (Table 1). Like NEEL19 V^+^, a significant percentage increase of around 6.85% in 3 µL, 14.95% in 4 µL, and 11.55% in 5 µL of stomatal length and 14.19% in 3 µL, 18.20% in 4 µL, and 19.34% in 5 µL of width was observed.

## 4. Discussion

This study identified the organic and inorganic volatiles of phylloplane isolate NEEL19, which were emitted and studied to determine whether any of them interact beneficially with mung bean and fenugreek seedlings in an in vitro assay. The phyllosphere microbiome comprises a wide range of beneficial bacteria [28,40]. To demonstrate high saprophytic competence in the rhizosphere, a phyllosphere strain requires certain characteristics, such as siderophores and antimicrobial compounds. Additionally, phyllosphere strains need to quickly metabolize the organic compounds (root exudates) to effectively compete with soil and root-adapted microbes [41,42]. Although using phyllosphere-associated bacteria directly for plant growth is challenging, it is suggested that direct use of phyllosphere-associated bacteria releasing volatiles could be a viable alternative. In that context, we previously demonstrated that the tea plant phylloplane isolate NEEL19 can produce siderophore and indole-3-acetic acid, as well as intriguingly generate phytohormones by sensing and metabolizing 1-Octanol, a plant volatile organic compound [43]. Additionally, this investigation revealed another PGP trait of NEEL19, i.e., phosphate solubilization. As demonstrated by the latest studies, PGPR-released volatiles have the potential to increase plant growth without direct contact while promoting plant tolerance to biotic and abiotic stresses [44]. It is also observed that, without physical contact, NEEL19 V^+^ significantly induced shoot and root length, and increased the lateral roots of mung bean seedlings cultivated in cotton beds in PPD under saline stress. On the other hand, NEEL19 V^+^ stimulates the biomass and shoot length of fenugreek seedlings. These results indicate that the bioactive volatiles were synthesized by phyllosphere-residing NEEL19 in sufficient quantities to interact and induce growth, along with saline tolerance of plants. Bacteria have the inherent capacity to generate and release a variety of organic and inorganic volatile metabolites, significantly impacting plant development [9]. Such findings are consistent with a previous report that VOCs of *Pseudomonas* sp. enhance both shoot and root length in mung bean seedlings [39]. Likewise, *P. putida* SJ46, emitting VOCs, promotes shoot and root growth in *Mentha piperita* under saline stress and also mentioned that, volatile exposure of *Alcaligenes faecalis* increased expression of the auxin and gibberellin pathway in both shoot and root cells of *A. thaliana* [44]. Additionally, our results illustrate that mung beans treated to NEEL19 V^+^ enhanced their total chlorophyll content even under saline stress, and the overall chlorophyll content of fenugreek increases more. producing outcomes comparable to those seen with *P. pseudoalcaligenes* volatiles in boosting the synthesis of photosynthetic pigments, and this volatile substance contributes to drought tolerance in maize plants [45]. VOCs emitting *B. subtilis* induce abscisic acid expression in *A. thaliana*, resulting in raised chlorophyll content [46,47]. The understanding is still lacking, nevertheless, on how bacterial volatiles affect photosynthesis, phytohormones, and the expression of related genes. According to our research, NEEL19 volatiles show several similar responses (shoot length and chlorophyll enhancement) in both mung bean and fenugreek seedlings, whereas distinct impacts on root formation with comparable differences. Our findings suggest that BVC effects are dependent on plant species or variety, which is clarified by prior descriptions, and the use of a negative control demonstrated that volatiles from *E. coli* DH5*α* did not promote the growth of *A. thaliana* and tobacco plants in a co-culture setting [48,49]. Despite this, VOCs emitted by *E. coli* DH5*α* were found to enhance the biomass, secondary roots, and root hair length of *A. thaliana* plants, along with the biomass of both shoots and roots in co-cultivated rice [12,40]. However, the mode of action of bacterial volatile substances is highly dependent on the species of plant. A variety of circumstances and related factors influence the volatile-based interaction between bacteria and plants. For example, the impacts on plants can change depending on the bacterial culture conditions, such as experimental model, growth media, pH, temperature, oxygen levels [50,51,52], inoculum density, and development stage [53,54].

GC-MS analysis revealed a VOCs profiling of NEEL19, especially that holds sulfurized compounds, wherein DMDS is identified as the predominant compound released by NEEL19. The production of sulfur-containing volatiles, including sulfur dioxide, methanethiol, and dimethyl trisulfide, was notably impeded by salinity. Additionally, the synthesis of DMDS was comparatively reduced, while 2-butanone, 2-pentanone, 2-undecanone, and 1-tetradecene were synthesized, and various compounds were generated in NEEL19. These results demonstrate that the VOC profile changes when particular growth medium components are changed. Similar results from another study showed that endophytic *Pseudomonas aeruginosa* that emit DMDS decrease when exposed to saline stress [55]. Methionine or cysteine breaks down to create DMDS, and several bacterial species, including *Pseudomonas*, *Bacillus*, *Serratia*, and *Stenotrophomonas*, have been shown to emit DMDS, and these species have antifungal properties, affect mosquito behavior, and, depending on their DMDS concentration, promote plant growth [56,57,58]. Particularly, prior research has shown that *Burkholderia pyrrocinia* and *Paraburkholderia phytofirmans*, which emit major VOCs like DMDS and 2-undecanone, respectively, have been linked to the induction of salt tolerance in *A.thaliana* [14]. Based on this, we considered that NEEL19, which emits DMDS and 2-undecanone, is the major VOC influencing salt stress responses in mung bean seedlings in this study, though deeper research is still needed. DMDS exhibits the ability to induce various effects on different plant species, contingent upon their concentration. When 1.0 mg of DMDS was administered, tobacco growth was stimulated [37]. The expression of early response genes associated with the signaling of this phytohormone is likely to rise after DMDS modifies auxin levels [57]. The growth of *A. thaliana* was shown to be decreased by 0.471 mg of DMDS in the study conducted by Kai and Piechulla [20]. Unlike hydrogen sulfide, another sulfur-containing volatile that is directly integrated into cysteine and can also be the only sulfur supply for plant growth and the mechanism of DMDS assimilation in plants is uncertain [58]. While most of the prior research has concentrated on the VOCs produced by bacteria, our study also examined the likely VOCs and probable VICs released by NEEL19. Major VICs such as NH_3_, CO_2_, and (NH_4_)_2_CO_3_ were found using the trapping approach. At ambient temperature and atmospheric pressure, NH_3_-mediated CO_2_ capture takes place, resulting in the formation of CO_2_-containing ammonium salts, including ammonium bicarbonate (NH_4_HCO_3_), ammonium carbonate ((NH_4_)_2_CO_3_·H_2_O), and ammonium carbamate (NH_2_COONH_4_) [59,60]. In addition to lowering the amount of NEEL19 VOCs, salinity also significantly decreased emissions of VICs, but alkaline pH remained constant. VICs also actively takes part in the interactions between microorganisms and plants that are mediated by volatiles. Generally, bacteria emitting NH_3_ and CO_2_ directly or indirectly promote their host plants’ growth [25,61,62]. Conversely, Weise, et al. [63] indicated that *Serratia odorifera* 4Rx13 negatively affects *A. thaliana* plants by alkalizing their media through NH_3_ emissions. Therefore, in the sealed petri dish, a large concentration of such volatiles is likely to impede plant growth or may lead the way to necrosis. But other research found that *B. amyloliquefaciens* BF6′s VOCs can improve unfavorable conditions, like alkaline surroundings, and raise *A. thaliana*’s total chlorophyll content by facilitating the uptake of iron [64]. Thus, when a volatile combines with another volatile or chemical substance, its impact on plants may exhibit varying responses. Ammonium bicarbonate was one of the most viable slow-release fertilizers utilized as C/N sources [65,66]. Moreover, lettuce plants treated with ammonium carbonate showed a considerable increase in total nitrogen content as well as biomass from both shoots and roots [67].

One of the identified VICs, NH_3_, was used for additional study and has been found to have a positive interaction with plants at lower concentrations (1–5 μL). This implied that the plant can sense and react to the volatiles, even at extremely low concentrations. According to this study, the vapor form of NH_4_OH induces a dosage-dependent increase in both shoot and root length in mung bean seedlings. Ammonia solutions at concentrations of 6, 9, and 12 mM have been shown to boost *maize* germination, shoot length, and root length, in line with the previous study [68], whereas *A. thaliana* growth has been reported to be inhibited by doses of 1 µmol or higher [69]. In addition, mung bean’s total chlorophyll content increased when exposed to NH_4_OH (3–5 μL), and significant differences in shoot and root length were also noted. Whilst fenugreek’s cotyledon showed a higher level of total chlorophyll when exposed to NH_4_OH (3–5 μL), but no significant changes in shoot or root length were noted. Our findings are in line with those of Sarasketa, et al. [69], who discovered that out of 47 wild accessions of *A. thaliana*, a 1 mM NH_4_^+^ supplement substantially reduced shoot fresh weight in 24 species, whereas Sanchez-Zabala, et al. [70] observed a significant increase in chlorophyll for all 47 types, but the difference varies between each type of species. This suggests that several individual plants of the same species showed a wide range of chlorophyll levels instead of a consistent reaction. In addition, both NEEL19 V^+^ and NH_4_OH exposure exhibited a congruent effect on the seed coat color of fenugreek, while no such change was observed in the seed coat of mung bean. Similarly, alterations in the seed coat color of fenugreek from the typical yellow to green were observed following induced chemical mutations through exposure to 0.06% methyl methane sulfonate [71]. These results show that the amount of NH_3_ can have either favorable or detrimental effects, depending on the type of plant. To further understand how and why the leaf pigment rises in relation to the N-source supplied, future research focusing on biogenic ammonia nourishment and its interaction with leaf pigment composition and biosynthesis-associated genes will be beneficial.

Bacterial VOCs can alter the physiological activity of plants by increasing stomatal conductance [44]. Alterations in stomatal size and density can be influenced by environmental factors [72]. According to a previous study, pH may indirectly affect the kinetics of stomatal growth, particularly in alkaline environments, where bigger stomata are seen [73]. Similar to this, NH_3_ released by NEEL19 causes the PPD to become alkaline. Thus, mung bean seedlings’ stomata increased in size and density in response to exposure to NH_3_ and NEEL19 V^+^. Research indicates that *A. thaliana*’s drought resistance is enhanced by VOCs emitted from *P. chlororaphis* O6, which promote stomatal closure [74]. Similarly, when exposed to NH_3_ and NEEL19 V^+^, mung bean stomatal closure was improved. Prior research indicates that mutations in specific genes and transcription factors result in the overproduction of meristemoids near existing stomata, consequently leading to contiguous stomatal clustering [75]. Stomagen, an intercellular signaling molecule, enhances the density of stomata in the apoplast and promotes photosynthesis. Stomatal clusters in adolescent leaves result from the overexpression of stomagen [76]. Similarly, mung bean seedlings exposed to NH_3_ and NEEL19 V^+^ displayed stomatal clusters known as contiguous stomata. In the leaf stomatal cluster of mung bean and the seed coat color variation of fenugreek, revealed in the NH_4_OH exposure assay, this finding coincides with the comparable morphological alterations of NEEL19 V^+^ treated seedlings under the PPD assay. Gaseous ammonia plays an important role in plant development by acting as a signaling molecule [68]. Particularly, the interaction between plants and the biogenic NH_3_ generated by bacteria is significantly influenced by the quantity, duration of exposure, and ammonia mixtures, such as ammonium carbonate or bicarbonate.

## 5. Conclusions

Our findings have shown that phylloplane isolate NEEL19 volatiles significantly stimulated seedling growth promotion and enhanced salt tolerance as well, and this study highlighted the impact of the interactions of biogenic ammonia which induce considerable growth enhancement and physio-morphological variability in their modes of action and the receptivity of individual plants. This experiment indicates that phyllosphere-associated bacteria also enhance plant development by emitting volatiles under in vitro conditions. However, phyllosphere’s biogenic volatile-emitting bacteria impacting the plant’s subterranean zone are still unidentified, which paves the way forward for more in vivo research.

## Figures and Tables

**Table 1:** Influence of the volatiles emitted by NEEL19 (NEEL19 V^+^ and NEEL19 V^−^) and NH_4_OH (1 to 5 µL) on leaf stomata of mung bean seedlings.

Treatment	Length of Guard Cell (µm)	Width of Guard Cell (µm)	Stomatal Density (n/mm^2^)	Closed Stomata (n/mm^2^)
NEEL19 V^−^	6.11 ± 0.18 ^b^	5.45 ± 0.34 ^a^	17.06 ± 0.81 ^a^	8.79 ± 0.60 ^b^
NEEL19 V^+^	6.50 ± 0.27 ^a^	5.73 ± 0.12 ^a^	19.81 ± 0.28 ^a^	17.32 ± 0.00 ^a^
5 µL ddH_2_O	5.68 ± 0.02 ^d^	4.87 ± 0.12 ^d^	18.63 ± 0.82 ^b^	9.32 ± 1.64 ^b^
1 µL NH_4_OH	5.78 ± 0.04 ^c,d^	5.30 ± 0.06 ^b,c^	17.59 ± 0.23 ^b^	8.92 ± 1.59 ^b^
2 µL NH_4_OH	5.72 ± 0.21 ^d^	5.05 ± 0.17 ^c,d^	18.77 ± 0.60 ^b^	9.97 ± 0.90 ^b^
3 µL NH_4_OH	6.07 ± 0.23 ^b,c^	5.55 ± 0.29 ^a,b^	22.18 ± 0.45 ^a^	14.70 ± 0.22 ^a^
4 µL NH_4_OH	6.53 ± 0.17 ^a^	5.75 ± 0.14 ^a^	21.13 ± 1.59 ^a^	14.70 ± 1.27 ^a^
5 µL NH_4_OH	6.34 ± 0.03 ^a,b^	5.80 ± 0.19 ^a^	20.73 ± 1.38 ^a^	14.44 ± 2.02 ^a^

About 5 µL of ddH_2_O was used as a control for the NH_4_OH assay. Significant differences (with and without NEEL19 volatiles treatments followed by Tukey’s test, *p* < 0.05) among treatments are indicated by letters. Error bars mean and significant differences (NH_4_OH vapor exposure analysis followed by Duncans test, *p* < 0.05) among treatments are indicated by letters (n = 41).

## Data Availability

The original contributions presented in this study are included in the article/Supplementary Material. Further inquiries can be directed to the corresponding authors.

## Supplementary Materials

The following supporting information can be downloaded at: https://www.mdpi.com/article/10.3390/microorganisms13122754/s1. Figure S1: (A) Growth analysis of NEEL19 under different salt concentrations (0, 2, 4, 6, 8, and 10% of NaCl) in the nutrient broth. (B) appearance of halo zone formation (arrow indicated) of mineral phosphate solubilization by NEEL19; Figure S2: Impact of mung bean seedlings growth in PPD after 7 d of exposure to: (A–F) NEEL19 volatiles under different saline conditions (0, 50, and 100 mM NaCl) and (G–L) aqueous NH_4_OH (0–5 μL) vapor. The circle indicates enhancement lateral root formation; Figure S3: Mass spectrometry data of plant growth benifical volatile organic compounds identified in NEEL19 under nutrient agar with and without supplementation of 100 mM NaCl; Figure S4: Morphological changes upon exposure to (A) NEEL19 V^−^, (B) NEEL19 V^+^ and (C−H). NH_4_OH (0–5 μL) on fenugreek seedling growth. A circle illustrates alterations in the hue of the seed coat; Figure S5: Impact of NEEL19 volatiles on fenugreek seedlings growth enhancement. (A) Biomass, (B) Shoot length, (C) Root length, (D) Chlorophyll a, (E) Chlorophyll b, and (F). Total chlorophyll a + b. Significant differences (ANOVA followed by Tukey’s test, *p* < 0.05) among treatments are indicated by letters (n = 3); Figure S6: Influence of the aqueous ammonia vapors (1–5 µL NH_4_OH) dose dependent growth analysis on fenugreek seedlings. (A) Fresh biomass, (B) Shoot length, (C) Root length, (D) Chlorophyll a, (E) Chlorophyll b and (F). Total Chlorophyll a + b. About 5 µL of ddH_2_O used as a control. Significant differences (ANOVA followed by Duncans test, *p* < 0.05) among treatments are indicated by letters (n = 3); Table S1: NEEL19 released VOCs profile under 0 mM and 100 mM NaCl concentrations.
