# Supplementary material for: Influence of Pseudomonas sp. NEEL19 Expelled Volatile Compounds on Growth and Development of Crop Seedlings
Source: Microorganisms. 2025 Dec 4;13(12):2754. doi: 10.3390/microorganisms13122754 (PMC12735227; doi:10.3390/microorganisms13122754)
Supplement: Supplementary file 1 [file microorganisms-13-02754-s001.zip › microorganisms-3991726-supplementary.pdf]

A

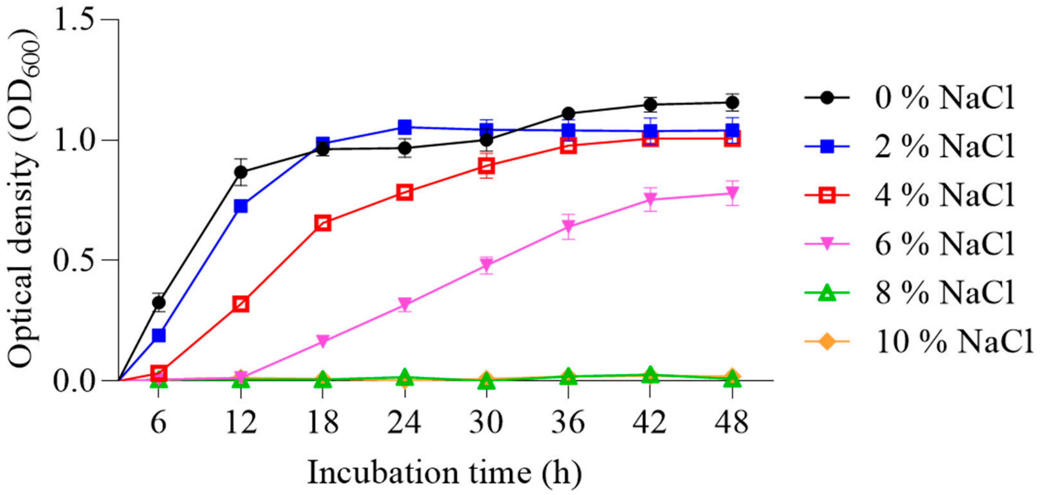

B

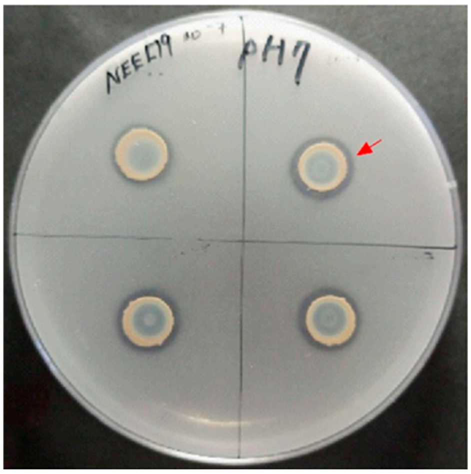

Figure S1. A. Growth analysis of NEEL19 under different salt concentrations (0, 2, 4, 6, 8, and 10 % of NaCl) in the nutrient broth. **B.** appearance of halo zone formation (arrow indicated) of mineral phosphate solubilization by NEEL19

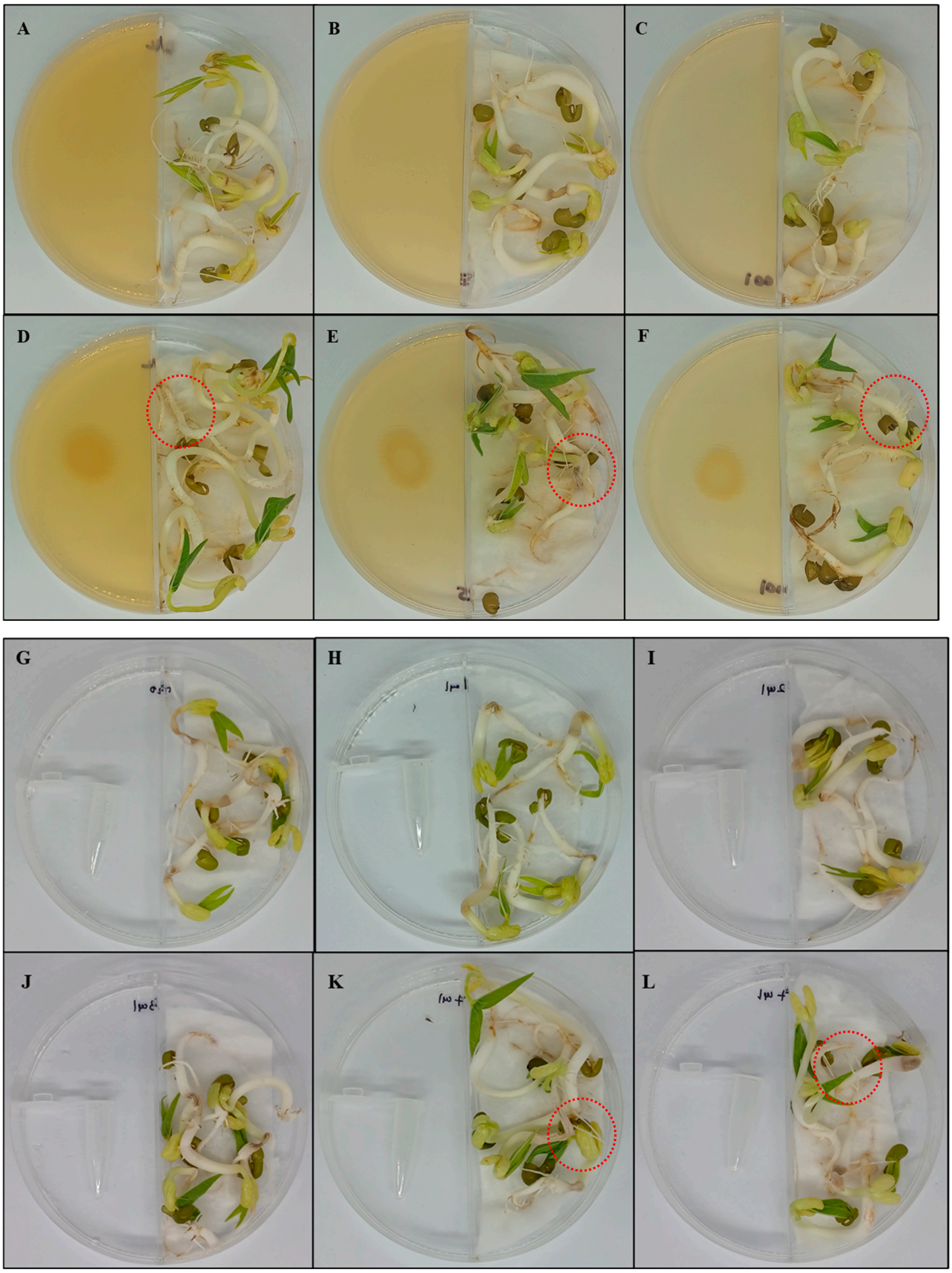

Figure S2. Impact of mung bean seedlings growth in PPD after 7 d of exposure to: **A**−**F** NEEL19 volatiles under different saline conditions (0, 50, and 100 mM NaCl) and **G**–**L** aqueous NH_4OH (0−5 μL) vapor. The circle indicates enhancement lateral root formation.

Table S1. NEEL19 released VOCs profile under 0 mM and 100 mM NaCl concentrations.

Peak No	Predicted VOCs	0 mM NaCl + NEEL19		100 mM NaCl + NEEL19		Activity	Reference
		Presence	Area (%)	Presence	Area (%)		
1	l-Alanine ethylamide, (S)-	+	0.51	−			[n.a]
2	Carbon dioxide	+	0.47	−		Induce plant growth	[20]
3	Sulfur dioxide	+	0.35	−			[n.a]
4	Methanethiol	+	0.19	−			[n.a]
5	2-Butanone	−		+	0.63	Enhancement of plant growth	[3, 16]
6	2-Pentanone	−		+	0.68	Enhancement of plant biomass	[10, 15]
7	Methyl thiolacetate	+	1.49	+	3.61		[n.a]
8	Methyl Isobutyl Ketone	+	0.49	+	0.68		[n.a]
9	Disulfide, dimethyl	+	61.75	+	32.78	Induce plant growth and antifungal activity	[14, 37]
10	Furan, 2-(methoxymethyl)-	−		+	1.12		[n.a]
11	Dimethyl trisulfide	+	1.22	−		Antifungal activity	[3]

12	Phosphonic acid, (p-hydroxyphenyl)-	−		+	2.62		[n.a]
13	1-Undecene	+	5.71	+	8.74	Antifungal activity	[38]
14	Cyclohexanol, 5-methyl-2-(1-methylethyl)-	+	0.5	−			[n.a]
15	2-Undecanone	−		+	0.49	Enhancement of leaf area and root length	[10, 14]
16	Cyclododecene, (Z)-	+	1.12	+	10.64		[n.a]
17	1-Tetradecene	−		+	4.44		[n.a]
18	Tetradecane	−		+	0.55		[n.a]
19	7-Tetradecene	−		+	0.59		[n.a]
20	(Z)6-Pentadecen-1-ol	−		+	0.68		[n.a]
21	2-Tridecanone	−		+	2.15	Induce s plant growth	[15]
22	11-Tetradecen-1-ol, (E)-	−		+	1.05		[n.a]
23	Nonadecane	+	0.99	−		Antifungal activity	[39]
24	Cyclooctasiloxane, hexadecamethyl-	−		+	0.97		[n.a]

25	Cyclononasiloxane, octadecamethyl-	−	+	1.66	[n.a]

Identical compounds found in both NEEL19 inoculated and non-inoculated NA (with and without 100 mM NaCl) media were not included. (+) presence; (−) absence; (n.a) information not available or unknown function. Peaks below a 90 % similarity index (SI) are removed using the NIST library.

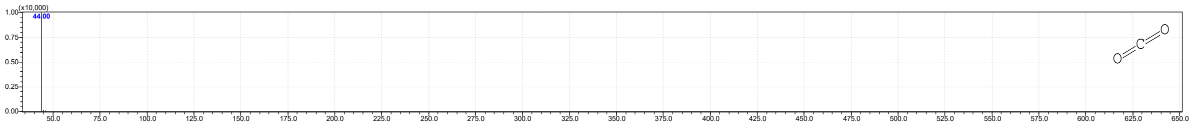

Peak No. 2. Carbon dioxide

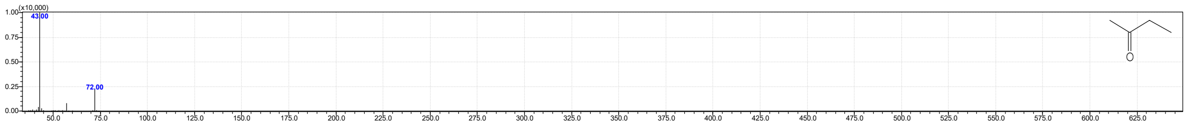

Peak No. 5. 2-Butanone

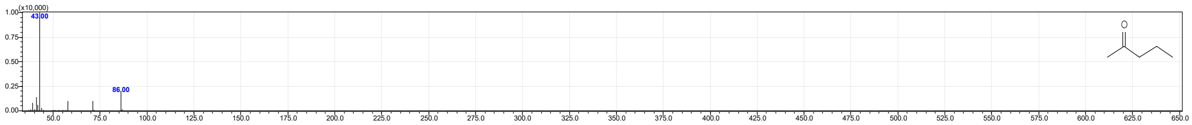

Peak No. 6. 2-Pentanone

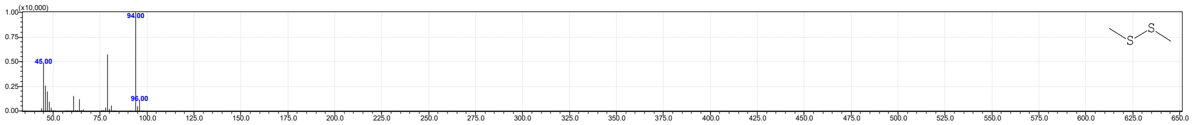

Peak No. 9. Disulfide, dimethyl

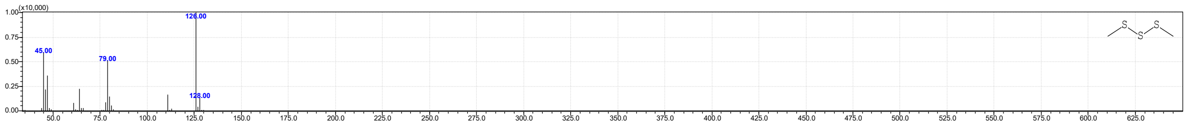

Peak No. 11. Dimethyl trisulfide

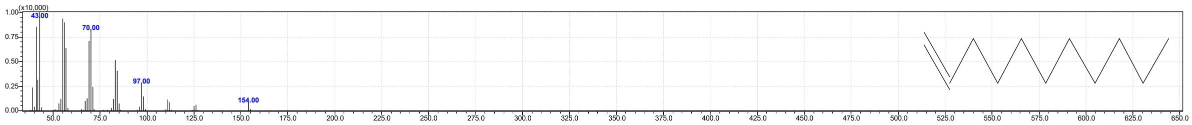

Peak No. 13. 1-Undecene

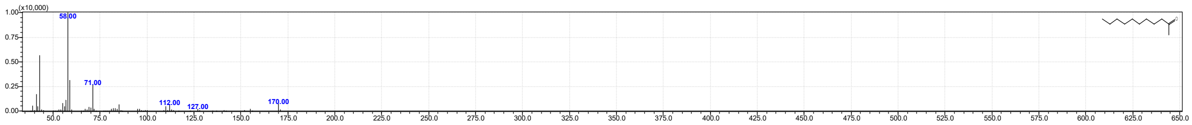

Peak No. 15. 2-Undecanone

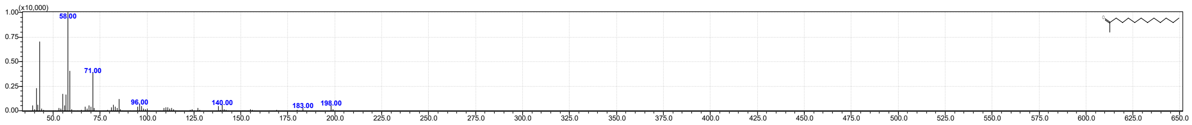

Peak No. 21. 2-Tridecanone

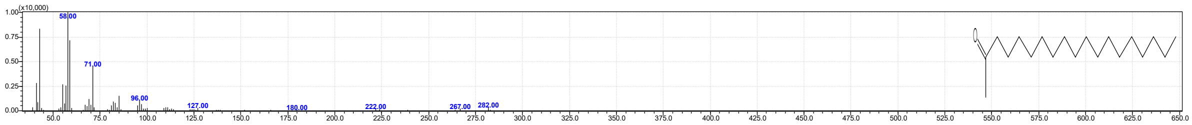

Peak No. 23. 2-Nonadecanone

Figure S3. Mass spectrometry data of plant growth benifical volatile organic compounds identified in NEEL19 under nutrient agar with and without supplementation of 100 mM NaCl.

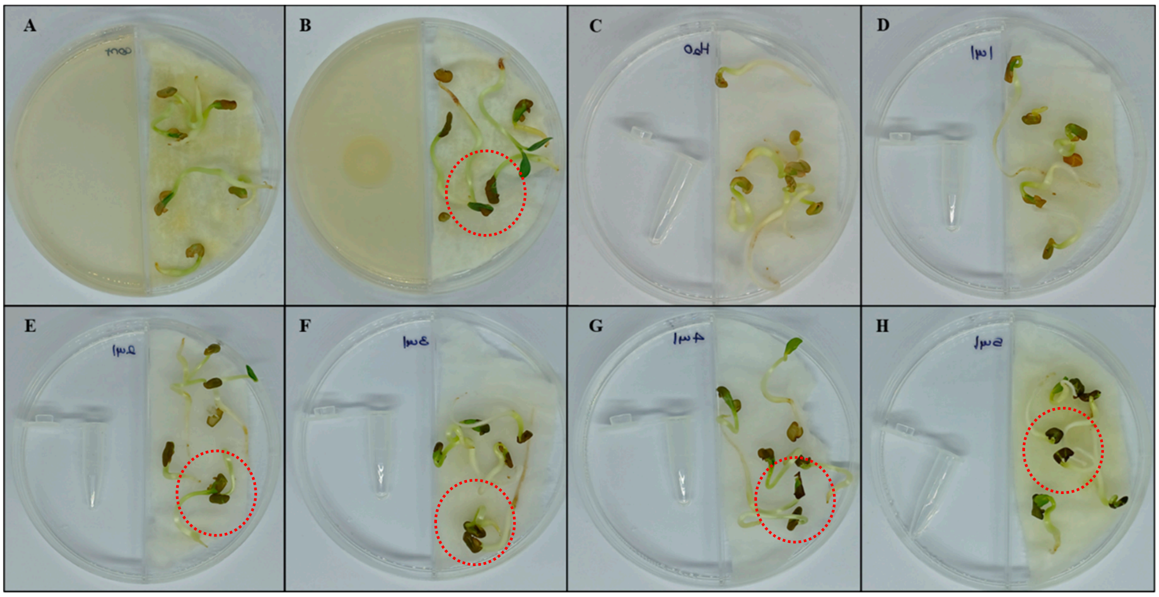

Figure S4. Morphological changes upon exposure to **A**. NEEL19 V^-, **B**. NEEL19 V^+ and **C−H**. NH_4OH (0−5 µL) on fenugreek seedling growth. A circle illustrates alterations in the hue of the seed coat.

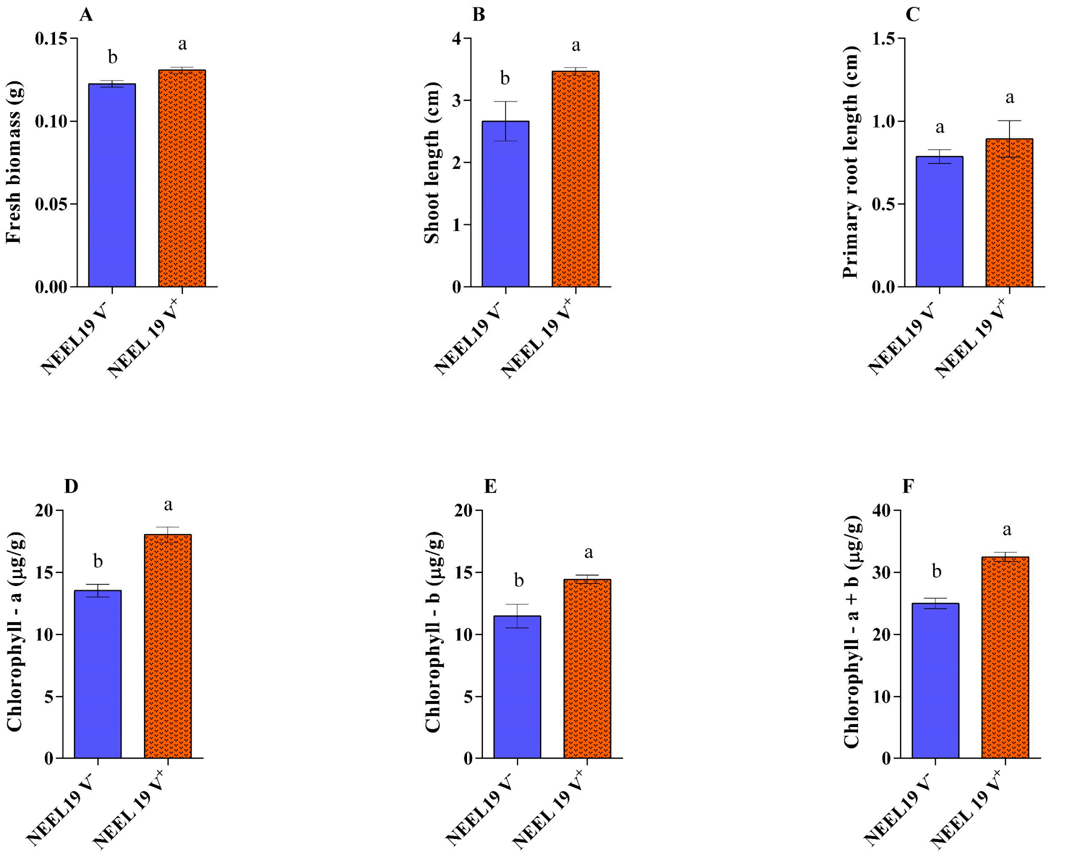

Figure S5. Impact of NEEL19 volatiles on fenugreek seedlings growth enhancement. **A**. Biomass, **B**. Shoot length, **C**. Root length, **D**. Chlorophyll a, **E**. Chlorophyll b, and **F**. Total chlorophyll a+b. Significant differences (ANOVA followed by Tukey's test, $p < 0.05$) among treatments are indicated by letters ($n = 3$).

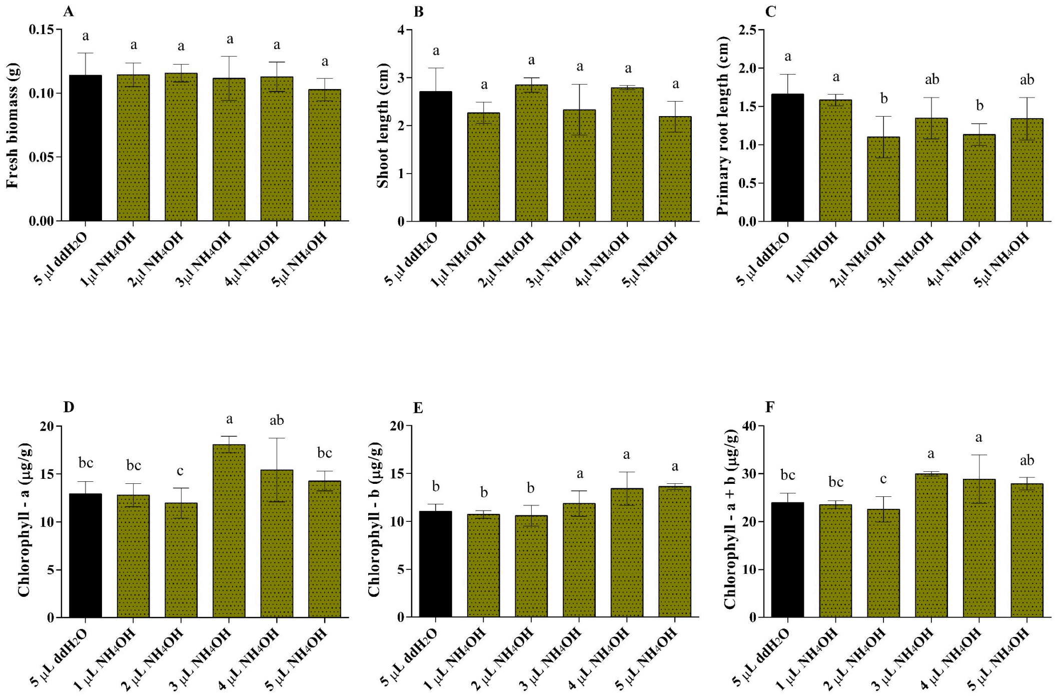

Figure S6. Influence of the aqueous ammonia vapors (1–5 µL NH_4OH) dose dependent growth analysis on fenugreek seedlings. **A**. Fresh biomass, **B**. Shoot length, **C**. Root length, **D**. Chlorophyll a, **E**. Chlorophyll b and **F**. Total Chlorophyll a+b. About 5 µL of ddH_2O used as a control. Significant differences (ANOVA followed by Duncans test, $p < 0.05$) among treatments are indicated by letters ($n = 3$).
